# Hydrothermally Synthesized CoS_2_ Nanostructures for Efficient and Recyclable Adsorptive Removal of Methylene Blue Contributing to Water Depollution

**DOI:** 10.1021/acsomega.5c08568

**Published:** 2025-12-18

**Authors:** Aziz Ait-Karra, Abdellah Mourak, Othmane Zakir, Rachid Idouhli, Burak Dikici, Abdesselam Abouelfida, Mohy Eddine Khadiri, Jaouad Benzakour

**Affiliations:** † Laboratory of Physical Chemistry of Materials and Environment, Faculty of Science Semlalia, 213489 Cadi Ayyad University, Bp 2390, Marrakech 40000, Morocco; ‡ Department of Mechanical Engineering, Faculty of Engineering, 37503 Ataturk University, Erzurum 25240, Turkey

## Abstract

The powder of cobalt disulfide (CoS_2_) was successfully prepared using the hydrothermal method at the temperatures of 150, 180, and 210 °C for 24 h. The series of characterization techniques, including SEM, EDX, XRD, and FTIR, confirmed the formation of pure CoS_2_ with a cubic crystal structure and an average crystallite size of about 14 nm, present in the form of spherical nanoparticles (NPs), whose diameters increased with increasing of the temperature. The adsorption activity of the CoS_2_ NPs was evaluated by using methylene blue (MB) dye. The study showed that all samples of CoS_2_ NPs are efficient adsorbents for MB under alkaline conditions (pH = 12). The kinetic study conducted on the most effective adsorbent obtained at 150 °C, representative of the synthesized CoS_2_ NPs, demonstrated that the pseudo-second-order is the kinetic model that describes the adsorption phenomenon. The analysis of the adsorption isotherms revealed that the process adopts the Freundlich model with a high correlation coefficient (*R* ^2^ = 0.99), indicating multilayer adsorption on a heterogeneous surface. The adsorption capacity increased with the temperature from 128.20 mg/g at 298 K to 222.22 mg·g^–1^ at 318 K, suggesting an endothermic process. The recyclability tests demonstrated that CoS_2_ NPs are stable and reusable adsorbents, showing a slight decrease in the MB removal efficiency, approximately 4%, after six successive reuses.

## Graphical abstract

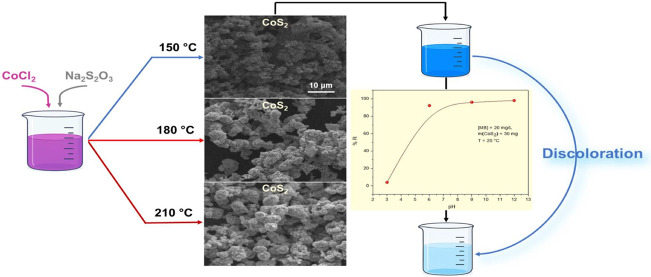

## 1 Introduction

Water pollution has become one of the most pressing environmental disasters of the 21st century, threatening ecosystems, human health, and global sustainability. Today, no one can deny that our world is experiencing a water crisis due to several reasons, including drought and the rapid growth of the population, which is accompanied by industrial expansion, intense demand for water in agriculture, and the irregular use of water, especially in swimming pools and golf courses. These reasons, among others, effectively participated in the pollution of water with distinct contaminants, including physical,^1^ organic and inorganic,^2^ biological,^3^ and radioactive^4^ substances. Among these water contaminants, organics have played an important role in the deterioration of water quality. Thus, all living beings will be badly impacted by the water they consume. This worldwide issue has prompted scientists to look for solutions that can mitigate it. Techniques such as adsorption^5,6^ and photocatalysis^7,8^ have been widely employed to remove organic pollutants from wastewater. Both techniques rely on material-based catalysts that exhibit unique electrical, optical, morphological, and surface properties, helping them in the removal of organic pollutants through physisorption, chemisorption, or photodegradation.^9^ Additionally, adsorption is generally less costly than photocatalysis, which requires light to activate the catalyst for enhanced degradation of organic contaminants.

Adsorption is an interfacial phenomenon that can occur between the catalyst surface and organic pollutant. It shows its applicability in the industrial sectors, contributing to the increase of their economy and efficiency.^10^ However, it was noticed that adsorption presents difficulties in the catalyst regeneration, which can increase its cost.^11^ Several catalysts have shown their effectiveness as adsorbents, such as carbon-based materials,^12^ clay materials,^13^ metal oxides,^14^ and metal sulfides.^15^ The application of metal sulfides in wastewater treatment through adsorption remains relatively underutilized compared with the aforementioned catalytic materials. However, metal sulfides have shown their potential to serve as promising alternatives for various application areas,^16,17^ particularly in wastewater treatment through the adsorption process.

In the last few decades, binary transition metal sulfides have emerged as potential catalysts for the elimination of organic colorants through an adsorption process. For instance, ZnS has successfully removed malachite green (MG) and methylene blue (MB) dyes.^18^ CuS has also demonstrated its effectiveness in adsorbing MB and bromophenol blue (BPB) dyes.^19^ A recent study demonstrated that NiS_2_–NiS nanocrystals can effectively adsorb MB dye.^20^ CdS nanoflowers have shown their ability to adsorb Rhodamine B (RhB) from artificially polluted water.^21^ These successes, among others, have prompted us to develop binary metal sulfides for wastewater treatment through the adsorption process. These findings highlight the versatility of transition metal sulfides as effective adsorbents, although further exploration is needed to realize their potential, especially for less-studied compounds.

In comparison with other metal sulfides, CoS_2_ materials have been known for their advantages, including low cost, high chemical stability, and excellent electrocatalytic performances.^22^ Moreover, the CoS_2_ materials have shown excellent electrical properties, including higher electronic conductivity,^23^ making them suitable candidates for catalytic applications such as adsorption.^24^ The nanostructured morphology of CoS_2_, typically composed of spherical grains with nanometric size, also provides a high specific surface area that facilitates efficient dye removal.^25^ These features distinguish CoS_2_ as a promising and innovative candidate for wastewater treatment applications. Despite the growing interest in metal sulfide adsorbents, as far as we are aware, previous studies have not focused on the preparation of CoS_2_ nanomaterials for adsorption applications. In this study, we report the elaboration of CoS_2_ nanoparticles (NPs) by using a simple and green hydrothermal method. The CoS_2_ NPs were then characterized and employed as adsorbents to remove the MB dye from artificially polluted water. MB was selected due to its extensive use in textile and chemical industries, its high stability and resistance to biodegradation, and its strong color that allows easy monitoring by UV–Vis spectroscopy. These factors make MB a representative dye for evaluating adsorption performance and enable straightforward comparison with previous studies. To understand the adsorption phenomenon of MB molecules on the active surfaces of CoS_2_ NPs, both kinetic and thermodynamic analyses were conducted. Finally, reusability tests were performed to evaluate the recyclability and stability of the CoS_2_ adsorbents. This study not only explores a novel application of CoS_2_ for dye adsorption but also contributes to the development of cost-effective and recyclable adsorbents for wastewater treatment.

## 2 Experimental Procedures

### 2.1 Materials

Cobalt chloride hexahydrate CoCl_2_·6H_2_O (99.5%), Sodium thiosulfate pentahydrate Na_2_S_2_O_3_·5H_2_O (99.0%), and methylene blue (MB) were acquired from VWR Chemicals (China), PanReac Química S.A. (Barcelona, Spain), and Sigma-Aldrich (St. Louis, USA), respectively.

### 2.2 Synthesis of CoS_2_ NPs

CoS_2_ nanoparticles (NPs) were synthesized via hydrothermal technique over a period of 24 h. In a classical procedure,^26^ 285.5 mg of CoCl_2_·6H_2_O and 297.8 mg of Na_2_S_2_O_3_·5H_2_O were dissolved collectively in 60 mL of distilled water contained in a Teflon beaker of 100 mL capacity and then vigorously stirred for 30 min. Subsequently, the beaker was put inside a sealed stainless-steel autoclave to undergo suitable heat treatment at three distinct temperatures (150, 180, and 210 °C). When the heat treatment was completed, the autoclave was allowed to cool in the oven. After that, a gray-black precipitate formed in the Teflon beaker was recovered through filtration accompanied by intense washing using distilled water and ethanol. Finally, the products were dried at 100 °C for 3–5 h.

### 2.3 Characterization of CoS_2_ NPs

In this work, samples of cobalt disulfide were analyzed using a series of characterization techniques. Scanning electron microscopy (SEM) was utilized to study the morphological properties of the CoS_2_ NPs, and the energy-dispersive X-ray spectroscopy (EDX) microanalysis was conducted to determine the elemental composition of the desired materials. The two techniques were performed using a TESCAN VEGA3 field emission scanning electron microscope (FESEM), operated at an accelerating voltage of 20 kV and under a pressure of 1.3 × 10^–4^ Pa. X-ray diffraction (XRD) was conducted to evaluate structural properties of CoS_2_ NPs, as well as to confirm their formation. The analysis was conducted using a Rigaku SmartLab SE X-ray diffractometer operating with Cu Kα radiation (wavelength 1.54184 Å) at 40 kV and 50 mA. The data were collected between 25° and 65° with a scan rate of 5°/min. Fourier transform infrared (FTIR) spectroscopy was conducted to determine the functional groups present in the samples, as well as to evaluate their affinity to water, using a VERTEX 70 FT-IR spectrometer with an ATR (attenuated total reflectance) attachment. The absorbance measurement was conducted using a PerkinElmer Fourier Transform 1720-X spectrophotometer operating with the wavelength 664 nm.

### 2.4 Adsorptive Performance of CoS_2_ NPs

The adsorption activity of CoS_2_ NPs was evaluated using a methylene blue (MB) water pollutant. The process was carried out in the dark with constant stirring at 250 rpm and room temperature. The effects of the parameters involved in the adsorption of MB by CoS_2_ NPs were studied under identical experimental conditions, and all experiments were performed in triplicate to highlight the reliability and reproducibility of the data.

The influence of adsorbent dose on the MB adsorption capacity was examined by mixing various amounts of CoS_2_, varying between 15 and 60 mg, with an MB solution of 20 mg·L^–1^. The mixture was stirred (250 rpm) at room temperature for 2 h, exceeding the adsorption equilibrium time that was required. The MB adsorbed quantity was calculated as reported below (eq 1).

The effect of pH on the adsorption activity of CoS_2_ NPs was investigated in acidic (pH 3), neutral (pH 7), and basic (pH 12) solutions. For this study, 30 mg of CoS_2_ was dispersed in an MB solution of 20 mg·L^–1^ and stirred at ambient temperature for 2 h.

Kinetic adsorption experiments were performed in a basic solution (pH 12) containing 30 mg of CoS_2_ dispersed in an MB solution of 20 mg·L^–1^, at two distinct temperatures of 298 and 318 K, under vigorous stirring at 250 rpm. At specific duration periods, samples of 5 mL were collected, centrifuged, and then analyzed using a PerkinElmer Fourier Transform 1720 spectrophotometer at a wavelength of 664 nm.

The efficiency of CoS_2_ NPs in removing MB dye from the wastewater was evaluated by calculating the removal percentage (*R*, %) and adsorption capacity (*q*_*t*_) using the following equations, respectively.^27^

1 $$R\left(\%\right)=\frac{C_{0}-C_{t}}{C_{0}}\times 100$$

2 $$q_{t}\left(\frac{mg}{g}\right)=\frac{C_{0}-C_{t}}{m}V$$

where *C*_0_ is the initial concentration of MB solution (mg·L^–1^), *C_t_* is its concentration (mg·L^–1^) at a time after the adsorption process, *m* is the dose of adsorbent (mg), and *V* is the volume of the MB solution (L).

For the study of adsorption isotherms, the dose of the CoS_2_ adsorbent was fixed at 30 mg, the MB concentration was varied between 20 and 80 mg·L^–1^, the pH of the solution was adjusted to 12, and the temperature was maintained at 298 and 318 K. The adsorption experiments were carried out in triplicate, and their isotherm data were analyzed with the linear forms of the Langmuir and Freundlich models. The Langmuir and the Freundlich equations are given by the formulas (eq 3)^20^ and (eq 4),^28^ respectively.

3 $$\frac{1}{q_{e}}=\frac{1}{q_{m}}+\frac{1}{K_{L}q_{m}C_{e}}$$

4 $$\ln\left(q_{e}\right)=\ln\left(K_{F}\right)+\frac{1}{n}\ln C_{e}$$

where *q_e_* is the equilibrium adsorption capacity (mg·g^–1^), *q_m_* is the maximum monolayer adsorption capacity (mg·g^–1^), *K*_L_ is the Langmuir constant related to the affinity of binding sites (L·mg^–1^), *C_e_* is the equilibrium concentration of MB in solution (mg·L^–1^), *K*_F_ is the Freundlich constant indicating adsorption capacity, and $\frac{1}{n}$ is the factor of heterogeneity indicating the adsorption intensity.

### 2.5 Regeneration of CoS_2_ NPs Adsorbents

The reusability of the CoS_2_ NPs adsorbents was evaluated under identical experimental conditions. After each use, the CoS_2_ NPs were recovered by centrifugation, followed by thorough washing with distilled water and ethanol solvents. The recovered solid was dried at 80 °C for 3 h.

## 3 Results and Discussions

The SEM micrographs of CoS_2_ nanoparticles synthesized at 150 °C (a, d), 180 °C (b, e), and 210 °C (c, f) are presented in Figure 1.

**Figure 1:**
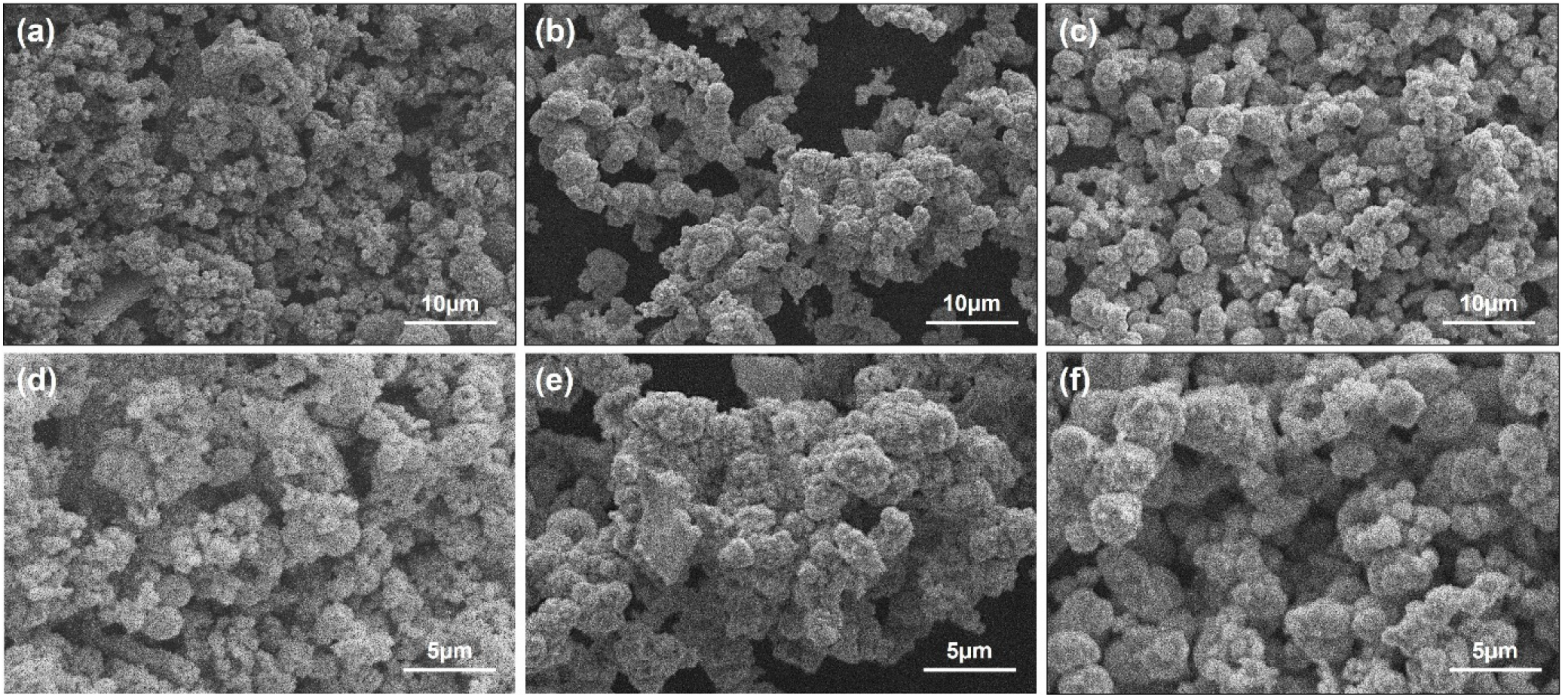
SEM images of hydrothermally synthesized CoS_2_ nanoparticles at different temperatures: (a, d) 150 °C, (b, e) 180 °C, and (c, f) 210 °C.

At lower magnification (Figure 1a–c), all samples exhibit aggregated spherical particles, with noticeable variations in grain size depending on the synthesis temperature. As the temperature increases, the particle size tends to grow, suggesting a reduction in the specific surface area. High-magnification images (Figure 1d–f) provide a closer view of the surface morphology, further confirming the temperature-dependent growth trend. Similar spherical morphologies were previously reported by Peng et al.^7^ for hierarchical CoS_2_ hollow nanospheres synthesized via a simple solution-based method. These observations indicate that the synthesis temperature plays a crucial role in tailoring the structural properties of CoS_2_ nanoparticles. These results clearly demonstrate that the synthesis temperature has a significant influence on the grain size of the CoS_2_ nanoparticles. As the temperature increases, the grain size becomes larger, which implies a corresponding decrease in the specific surface area.

The average grain diameter of the CoS_2_ nanoparticles was estimated using a classical approach by selecting ten clear and distinct grains from each SEM micrograph. The analysis revealed that the average grain diameters were approximately 681, 1468, and 2390 nm for the CoS_2_ materials obtained at 150, 180, and 210 °C, respectively. These results confirm that higher synthesis temperatures promote grain growth, most likely due to enhanced atomic diffusion and particle coalescence.

The SEM results demonstrated that temperature has a strong effect on the morphology of CoS_2_ nanoparticles, transforming them from smaller spherical grains to larger ones, which effectively influences their specific surface area and, consequently, their adsorption performance. Various parameters, such as reaction time, solvent nature, and the addition of surfactants, have been investigated to control the morphology of such materials. Recently, an emerging and innovative approach known as microfluidic synthesis has been introduced, offering precise control over particle size, shape, and uniformity during nanomaterial fabrication.^29–31^

The chemical composition of the synthesized materials was estimated by using energy-dispersive X-ray (EDX) analysis. The EDX spectra of the CoS_2_ materials obtained at 150 °C (a), 180 °C (b), and 210 °C (c) are presented in Figure 2. In all cases, only cobalt (Co) and sulfur (S) peaks were detected, confirming the chemical purity of the samples. Furthermore, the atomic percentage of sulfur was consistently higher than that of cobalt, indicating the successful formation of stoichiometric CoS_2_ and suggesting that excess sulfur may be adsorbed on the surface of the nanoparticles. Moreover, the atomic percentage of sulfur was observed to increase progressively with synthesis temperature, from 78.39% at 150 °C to 90.46% at 210 °C, suggesting that elevated temperatures may promote sulfur enrichment on the surface of the nanoparticles, potentially influencing their adsorption behavior.

**Figure 2:**
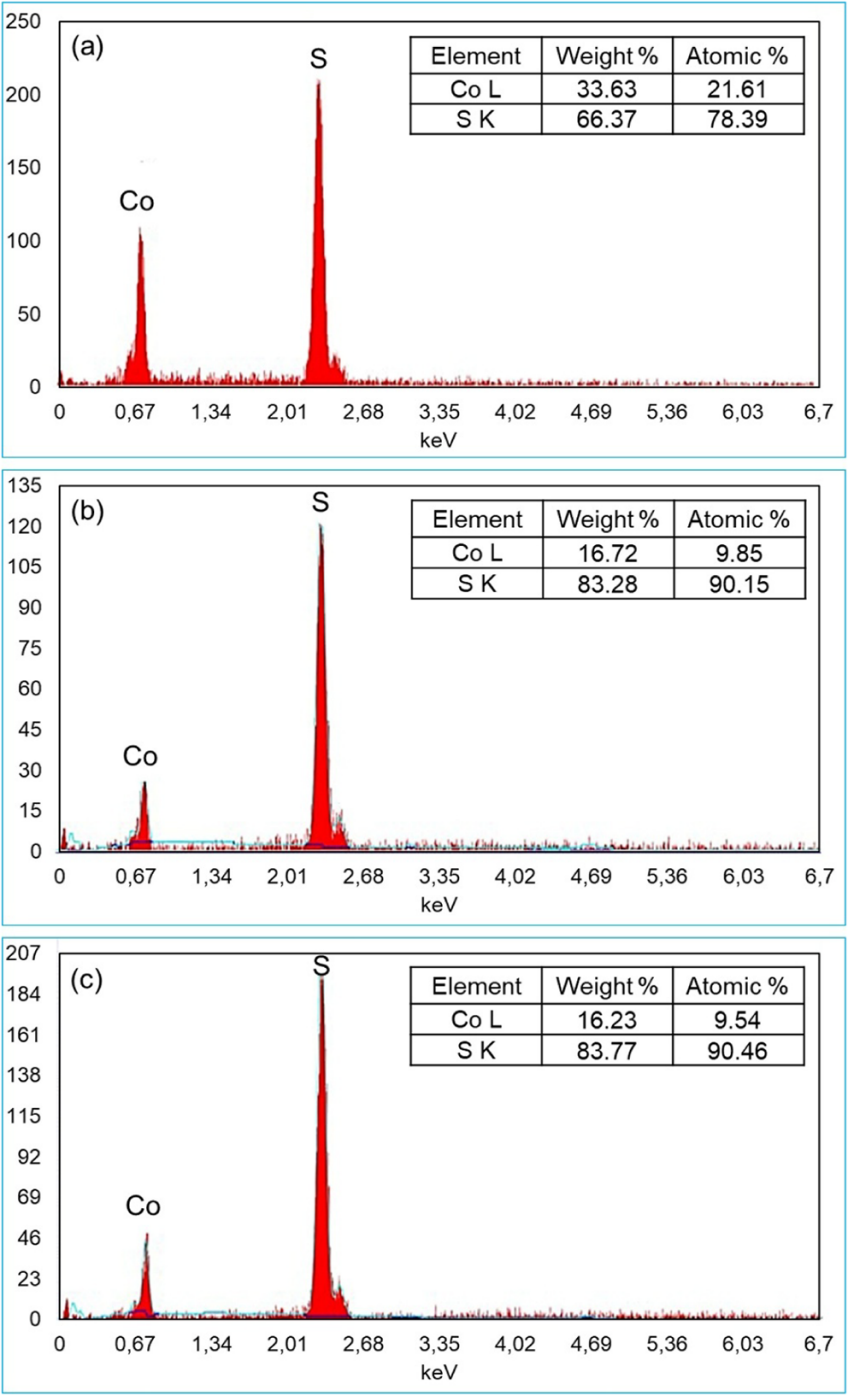
EDX spectra of CoS_2_ nanoparticles synthesized at different hydrothermal temperatures: (a) 150 °C, (b) 180 °C, and (c) 210 °C.

The XRD patterns of the CoS_2_ nanoparticles grown hydrothermally at 150 °C (a), 180 °C (b), and 210 °C (c) are presented in Figure 3. All samples exhibit distinct diffraction peaks at 2θ values of approximately 27.9°, 32.3°, 36.3°, 39.8°, 46.4°, 54.9°, 57.6°, 60.3°, and 62.7°, corresponding to the (111), (200), (210), (211), (220), (311), (222), (230), and (321) crystal planes of the cubic CoS_2_ phase. These diffraction features match well with the reference data from JCPDS Card No. 00-041-1471, confirming the successful formation of pure-phase CoS_2_.

**Figure 3:**
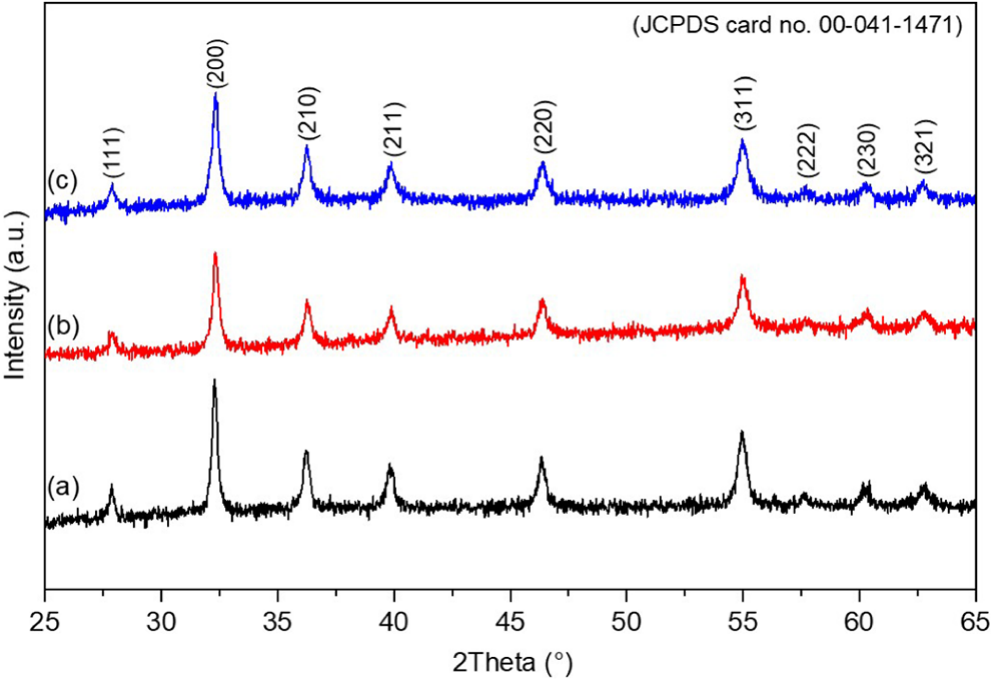
XRD patterns of CoS_2_ nanoparticles synthesized at different hydrothermal temperatures: (a) 150 °C, (b) 180 °C, and (c) 210 °C.

Additionally, no secondary or extraneous peaks were observed in the diffraction patterns, further confirming the phase purity of the synthesized CoS_2_ materials. Interestingly, the relative intensity of the diffraction peaks slightly decreased with increasing synthesis temperature, which may indicate a reduction in crystallinity at higher temperatures.^32^ This observation is consistent with previous XRD analyses of CoS_2_ materials reported in the literature.^33,34^

The main crystallite size (*D*) of the CoS_2_ samples was calculated from the full width at half-maximum (β) values of the most intense diffraction peaks, corresponding to the (200), (210), (211), and (311) crystal planes, using the Debye–Scherrer equation (eq 5).^35^ The calculated *D* values are summarized in Table 1.

5 $$D=\frac{0.9\times \lambda }{\beta \times \cos\theta }$$

where λ is the X-ray wavelength and θ is the Bragg angle.

**Table 1:** Crystallite Size and Lattice Parameters of CoS_2_ Nanoparticles Synthesized at Different Temperatures, Calculated from XRD Data Using the Debye–Scherrer Equation

Temperature (°C)	150	180	210
Crystallite size (nm)	13.893	14.455	13.618
Lattice parameter *a* (Å)	5.5464	5.5331	5.5364

Lattice parameters (LP) of the cubic unit cell were determined from the (200) diffraction peak using the standard crystallographic equation (eq 6).^26^ The calculated lattice parameter values are also presented in Table 1.

6 $$\frac{1}{d^{2}}=\frac{h^{2}+k^{2}+l^{2}}{a^{2}}$$

where, *h*, *k*, and *l* are the Miller indices, *a* is the lattice constant of the cubic unit cell, and *d* is the interplanar spacing.

The calculated lattice parameter *a* for all synthesized materials is in good agreement with the standard value (*a* = 5.5367 Å) reported in JCPDS Card No. 00-041-1471. As shown in Table 1, an inverse relationship was observed between crystallite size and the lattice parameter: as the crystallite size increased, the lattice parameter slightly decreased. This trend suggests that a smaller lattice parameter may indicate a more tightly packed crystal structure, potentially accommodating a greater number of unit cells within a given volume. Such packing could contribute to the observed increase in crystallite size.

The functional groups present in the synthesized CoS_2_ nanomaterials were identified by using Fourier-transform infrared (FTIR) spectroscopy. The FTIR spectra of CoS_2_ nanoparticles obtained at 150 °C (a), 180 °C (b), and 210 °C (c) are presented in Figure 4.

**Figure 4:**
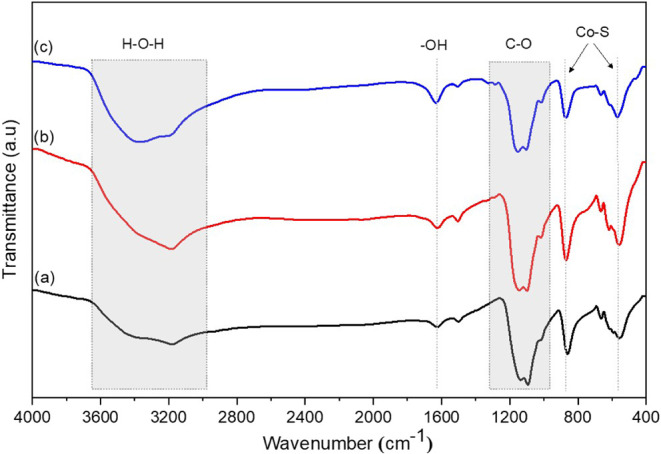
FTIR spectra of CoS_2_ nanoparticles synthesized at 150 °C (a), 180 °C (b), and 210 °C (c).

As shown in the figure, all samples exhibit similar transmittance bands, indicating a comparable surface chemistry. A large and intense absorption band in the wavenumber region of 2970–3630 cm^–1^ is likely assigned to O–H stretching and H–O–H bending vibrations, characteristic of H_2_O molecules adsorbed on the surface of the CoS_2_ nanoparticles.^35,36^ Additionally, a weak band around 1632 cm^–1^ matches H–O–H bending vibrations of molecular H_2_O or the deformation vibrations of surface hydroxyl (−OH) groups,^37^ further supporting the existence of adsorbed water on the material surface.

Another strong band observed in the wavenumber region of 950–1300 cm^–1^ is assigned to C–O bending vibrations, typically associated with alcohol groups.^38,39^ This feature may arise from residual ethanol used during the washing of the CoS_2_ nanoparticles or from acetone used to clean the sample holder. A medium-intensity band centered at 552 cm^–1^ is attributed to Co–S stretching vibrations, a characteristic signature of cobalt sulfide nanomaterials.^40,41^ Additionally, the absorption band around 875 cm^–1^ corresponds to another Co–S vibrational mode, which may be related to distinct bonding environments within the nanostructures.^26,37^ Overall, the FTIR analysis confirms the successful formation of CoS_2_ nanomaterials and highlights their pronounced hydrophilic nature, as evidenced by the strong O–H and H–O–H vibrational features.

As mentioned in the experimental section, CoS_2_ nanoparticles (NPs) were synthesized via a hydrothermal method using cobalt­(II) chloride (CoCl_2_) and sodium thiosulfate (Na_2_S_2_O_3_) as precursors. In aqueous solution, these reagents dissociate to release Co^2+^ and S_2_O_3_^2–^ ions, as shown in reactions (*R*1) and (*R*2).^42^

R1 $${\mathrm{CoCl}}_{2}\to {\mathrm{Co}}_{2}++2{\mathrm{Cl}}^{-}$$

R2 $${\mathrm{Na}}_{2}S_{2}O_{3}\to 2\mathrm{Na}++S_{2}O_{3}^{2-}$$

According to the literature, under hydrothermal conditions, thiosulfate ions (S_2_O_3_^2–^) can hydrolyze in the presence of water to produce hydrogen sulfide (H_2_S), which further ionizes to yield sulfide ions (S^2–^) and elemental sulfur (S),^43–45^ in accordance with the following reactions.

R3 $$S_{2}O_{3}^{2-}+H_{2}O\to H_{2}S+{\mathrm{SO}}_{3}^{2-}$$

R4 $$H_{2}S\to 2H^{+}+S^{2-}$$

The Co^2+^ ions then react with the S^2–^ ions to form cobalt monosulfide (CoS), as shown in the following reaction (*R*5):^26^

R5 $${\mathrm{Co}}^{2+}+S^{2-}\to \mathrm{CoS}$$

Subsequently, initially formed CoS reacts with elemental sulfur (S) to yield cobalt disulfide (CoS_2_) (*R*6):

R6 $$\mathrm{CoS}+S\to {\mathrm{CoS}}_{2}$$

In summary, CoS_2_ nanoparticles can be hydrothermally synthesized at temperatures above 150 °C through the overall reaction pathway represented below:

R7 $${\mathrm{CoCl}}_{2}+{\mathrm{Na}}_{2}S_{2}O_{3}+H_{2}O\to {\mathrm{CoS}}_{2}+\mathrm{NaCl}+\mathrm{byproducts}$$

As previously described, three CoS_2_ nanomaterials were successfully synthesized under different hydrothermal conditions. SEM analysis revealed that the sample synthesized at 150 °C exhibited smaller grain sizes compared with the other two samples. To determine the most effective adsorbent among the synthesized CoS_2_ materials, preliminary adsorption experiments were conducted using methylene blue (MB) solutions with an initial concentration of 20 mg·L^–1^, pH adjusted to 12, and 30 mg of CoS_2_ adsorbent at 25 °C.^26^ The adsorption process was carried out for 2 h.

From Figure 5, the adsorption capacity of the CoS_2_ samples synthesized at 150 °C (CS1), 180 °C (CS2), and 210 °C (CS3) was found to be 26.15, 25.70, and 23.53 mg g^–1^, respectively. The capacity of CS1 was slightly higher than that of CS2 and notably greater than CS3, confirming superior performance of CS1 under the tested conditions.

**Figure 5:**
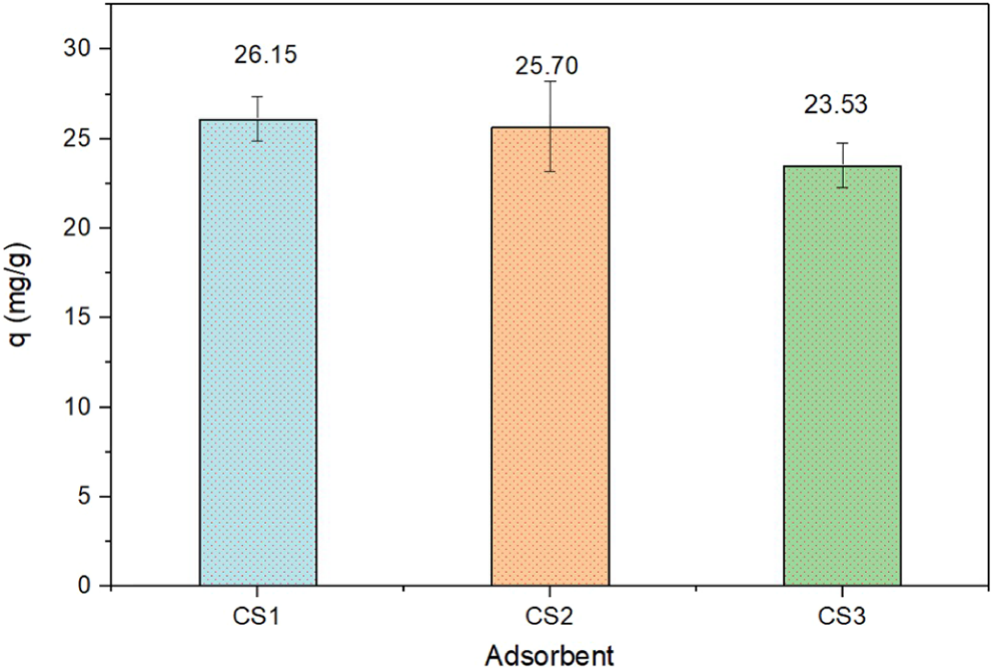
Adsorption capacities of CoS_2_ nanoparticles synthesized at 150 °C (CS1), 180 °C (CS2), and 210 °C (CS3) for methylene blue (MB) removal after 2 h of contact time.

This enhanced performance can be due to the smaller grain size of CS1, which offers a larger specific surface area (SSA) and thereby increases the available active sites for MB adsorption. Based on these results, the sample synthesized at 150 °C was selected as the representative CoS_2_ adsorbent for subsequent adsorption studies.

The effect of adsorbent mass on the MB removal was investigated to determine the optimal dose of CoS_2_ required for maximum adsorption of a fixed MB concentration of 20 mg·L^–1^. The experiments were conducted under identical conditions, with the CoS_2_ adsorbent mass varied from 15 to 60 mg. The equilibrium adsorption capacity (*q_e_*) was evaluated and is presented in Figure 6.

**Figure 6:**
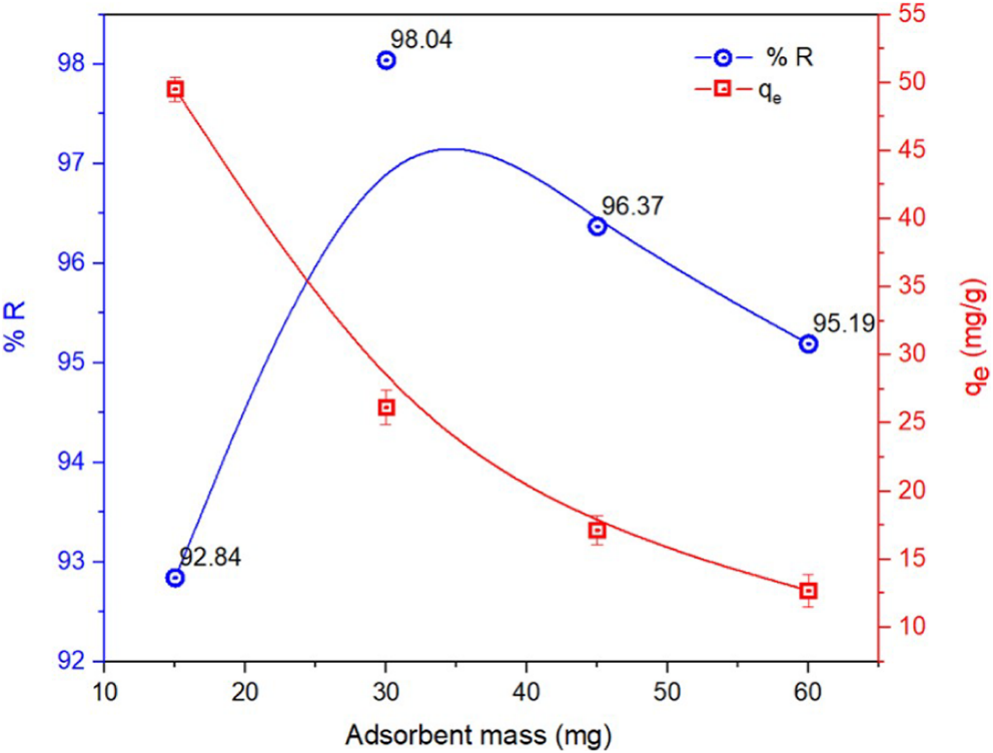
Effect of CoS_2_ nanoparticle dosage on the removal of methylene blue (MB) (left *y*-axis: MB removal efficiency and right *y*-axis: equilibrium adsorption capacity).

As observed in the figure, qe decreased with an increasing CoS_2_ dose. This trend can be justified by various factors. First, increasing the adsorbent dosage provides more available surface area and active sites, but since the MB concentration remains constant, the dye molecules are distributed across more particles, leading to a lower amount adsorbed per gram of adsorbent. Second, at higher doses, particle aggregation may occur, which reduces the effective surface area by blocking active sites.^46^ In addition, a higher adsorbent amount can reduce the driving force of mass transfer due to a lower concentration gradient between the solid and liquid phases.^47^

To select the most suitable CoS_2_ dosage for further experiments, the MB removal efficiency (%R) was also calculated and is plotted in Figure 6. The results showed that an adsorbent dose of 30 mg achieved the highest removal efficiency (98.04%) within 2 h. This can be explained by the fact that a dose below 30 mg provides insufficient surface area, while increasing the dose beyond 30 mg leads to a decline in efficiency due to aggregation-induced site blockage.^46,48^

The effect of solution pH on the adsorption activity of CoS_2_ nanoparticles was studied by dispersing 30 mg of the adsorbent in 40 mL of MB solution with a concentration of 20 mg·L^–1^. The pH was adjusted to 3, 6, 9, and 12 using dilute HCl or NaOH solutions. Figure 7 illustrates the evolution of the MB removal rate (%R) with solution pH.

**Figure 7:**
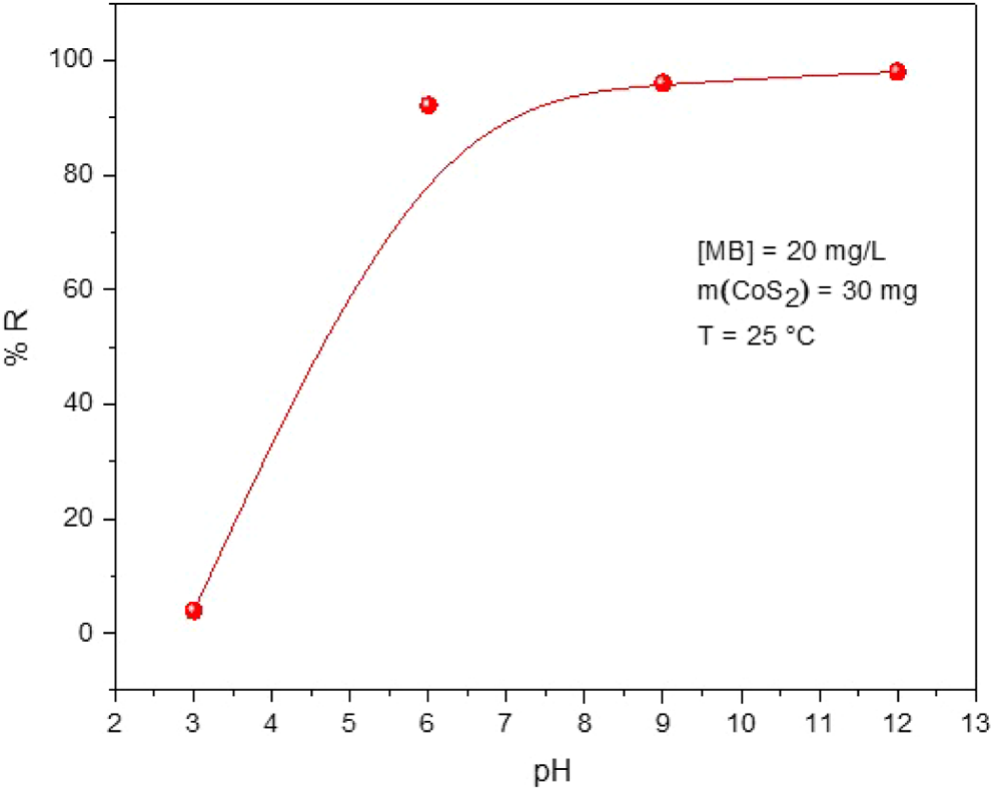
Effect of initial pH on the removal efficiency of MB by CoS_2_ nanoparticles.

As shown in Figure 7, the %R elevated progressively with increasing pH. This trend has been widely reported in the literature for MB adsorption by various adsorbents, including defatted *Carica papaya* seeds,^47^ teak tree bark powder (TTBP),^49^ Moroccan clays,^46^ and clay minerals such as smooth clay (SC), rigorous clay (RC), and bentonite clay (BC),^28^ among others.

This behavior can be due to the surface charge of the adsorbent and its resulting electrostatic interactions with MB molecules. At lower pH values, the surface of the CoS_2_ nanoparticles becomes positively charged owing to the presence of excess protons (H^+^) from the added acid used to adjust the pH. This leads to electrostatic repulsion between the cationic MB molecules and the positively charged CoS_2_ surface, consequently reducing the adsorption efficiency. As the pH increases, hydroxide ions (OH^–^) from the added base increase the negative charge on the surface of the CoS_2_ NPs. This promotes electrostatic attraction between the negative CoS_2_ surface and the positively charged MB molecules, resulting in enhanced adsorption performance.^50^

The maximum MB removal performance (98.04%) was observed at pH 12. This finding aligns well with previous reports on MB adsorption using other materials such as pyrophyllite,^46^ *Carica papaya* seeds,^47^ teak tree bark powder (TTBP),^49^ purified bentonite clay (PBC),^28^ and zeolites.^50^ This behavior can be explained by the point of zero charge (PZC) of CoS_2_, determined to be around pH 7.5. Below this pH, the surface of CoS_2_ is positively charged, leading to electrostatic repulsion with cationic MB molecules. Above this value, the surface becomes negatively charged, which promotes strong electrostatic attraction with MB and results in significantly higher adsorption efficiency.

To assess the rate-controlling mechanism of MB adsorption onto CoS_2_ nanoparticles, kinetic modeling was performed using the pseudo-first-order and pseudo-second-order models. The experiments were conducted under the following optimal conditions: MB concentration = 20 mg/L, pH = 12, adsorbent dose = 30 mg, and bath temperatures of 25 °C and 45 °C. Figure 8 illustrates the evolution of the MB color during the adsorption process. As evidenced by this figure, the color of the MB solution gradually changed over time from a blue solution to a colorless one, indicating the adsorption of MB molecules onto the active surface of CoS_2_ NPs adsorbent at 25 °C. This finding suggests a strong adsorption activity of CoS_2_ toward cationic dyes, particularly MB.

**Figure 8:**
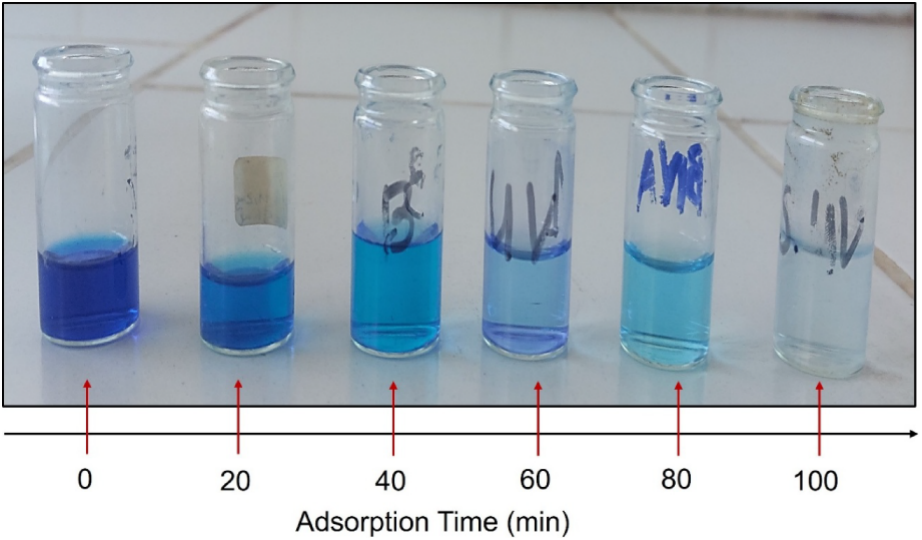
Illustrative photograph indicating the evolution of the color of the MB solution during adsorption onto CoS_2_ NPs at 25 °C.

The pseudo-first-order (PFO) kinetic model is given by its linearized form (eq 14),^35^ which relates the logarithmic difference between the adsorption capacity at the equilibrium (*q_e_*) and the adsorption capacity at time *t* (*q_t_*) to the contact time:

7 $$\ln\left(q_{e}-q_{t}\right)=\ln q_{e}-k_{1}t$$

where, *k*_1_ is the rate constant of the PFO model.

The linear fitting of the pseudo-first-order kinetic model is presented in Figure 9a. As observed, the experimental variation of ln­(*q_e_* – *q_t_*) with time shows noticeable deviation from the theoretical fitting curves, resulting in correlation coefficients (*R* ^2^) of 0.908 and 0.916 for 25 °C and 45 °C, respectively. These moderate *R* ^2^ values indicate that the pseudo-first-order model fails to accurately describe the adsorption kinetics of MB onto CoS_2_ nanoparticles, suggesting that physisorption is not the dominant mechanism. This observation is consistent with previous studies on MB adsorption using GO-MNP composites^27^ and sepiolite adsorbents.^51^ Furthermore, the calculated equilibrium adsorption capacities (*q*_cal_) derived from the model are relatively close to the experimentally obtained values (*q*_exp_), suggesting some partial agreement. The *q_e_* and the rate constant *k*_1_ values were calculated from the slope and intercept of the fitted plots, and the corresponding kinetic parameters are summarized in Table 2.

**Figure 9:**
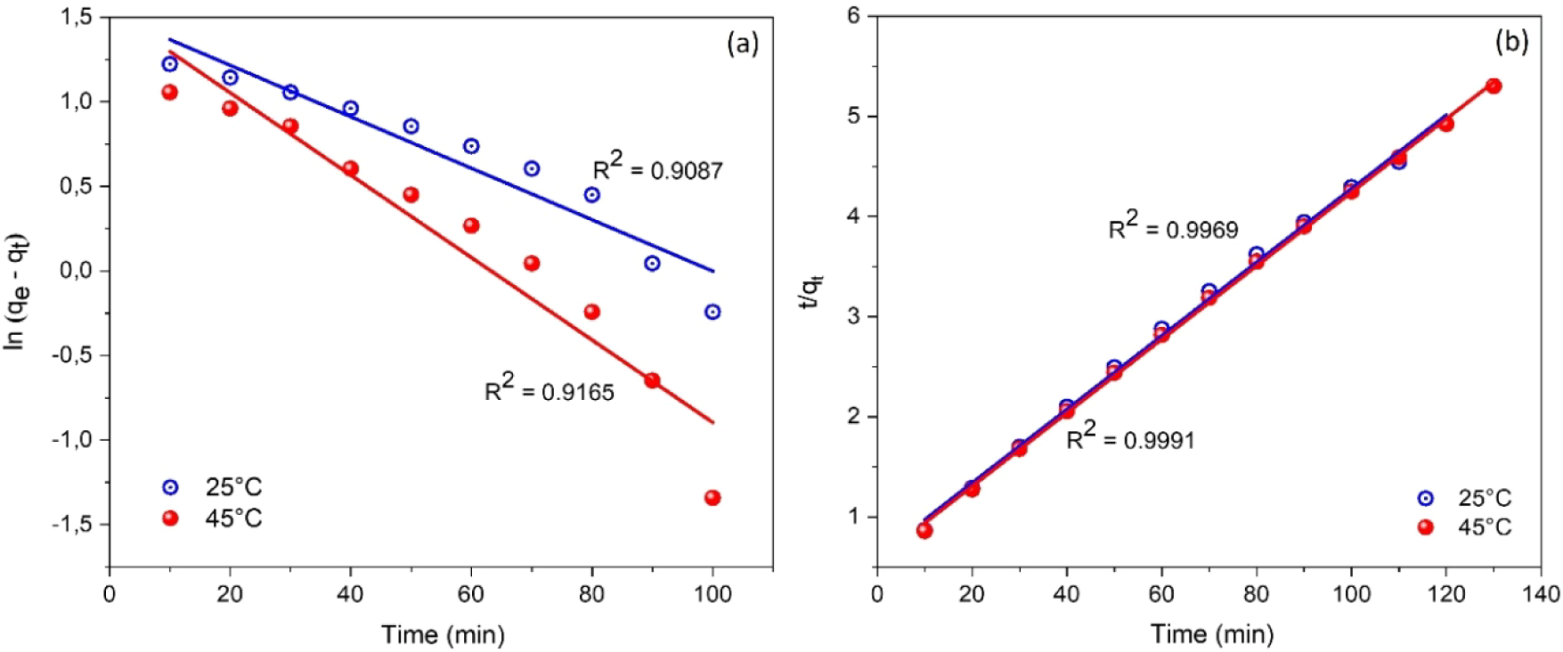
Kinetic modeling of MB adsorption onto CoS_2_ nanoparticles: (a) pseudo-first-order and (b) pseudo-second-order kinetic plots at two different temperatures (25 °C and 45 °C).

**Table 2:** Kinetic Parameters for the Adsorption of Methylene Blue (MB) onto CoS_2_ Nanoparticles Obtained from the Pseudo-First-Order and Pseudo-Second-Order Models at 25 °C and 45 °C

Kinetic model	Pseudo-first-order			Pseudo-second-order			
Kinetic parameters	*k*_1_ (min^–1^)	*R* ^2^	*q*_cal_ (mg/g)	*k*_2_ (g/mg·min)	*R* ^2^	*q*_cal_ (mg/g)	*q*_exp_ (mg/g)
*T* = 25 °C	0.0152	0.908	4.5735	0.002223	0.996	27.233	26.15
*T* = 45 °C	0.0243	0.916	4.6681	0.002371	0.999	27.115	26.40

The pseudo-second-order (PSO) kinetic model is represented by a linearized form that involves plotting $\frac{t}{q_{t}}$ against time (*t*), as expressed in eq 15.^35^

8 $$\frac{t}{q_{t}}=\frac{1}{k_{2}q_{e}^{2}}+\frac{t}{q_{e}}$$

where *k*_2_ is the rate constant of the PSO model.

The experimental data and corresponding fitting results for this model are shown in Figure 9b. The figure clearly shows that the PSO model accurately describes the adsorption process, as indicated by the excellent agreement between experimental and calculated data and correlation coefficients (*R* ^2^) exceeding 0.99 at both 25 °C and 45 °C.

Additionally, the calculated equilibrium adsorption capacities (*q*_cal_) obtained from the PSO model closely match the experimental values (*q*_exp_), confirming the model’s reliability. This strong correlation indicates that the adsorption of MB onto CoS_2_ nanoparticles follows pseudo-second-order kinetics.

The applicability of the PSO model implies that the adsorption mechanism is primarily governed by chemisorption, associated with valence forces through electron sharing or exchange between MB molecules and active sites on the CoS_2_ surface. These findings are consistent with previous reports on MB adsorption by CuS,^35^ as well as CoS and Co_9_S_2_ adsorbents,^26^ further supporting that MB adsorption onto CoS_2_ nanoparticles proceeds via a chemisorption mechanism. The values of the parameters *q_e_* and *k*_2_ were calculated and are summarized in Table 2.

To understand the nature and mechanism of MB adsorption onto the CoS_2_ adsorbent, adsorption capacity was evaluated using the Langmuir and Freundlich isotherm models. Analysis of the Freundlich model parameters, particularly the adsorption capacity constant *K*_F_, and the parameters *n* and 1/*n*, indicated that MB adsorption on CoS_2_ is favorable, as evidenced by the *n* value greater than 1. This suggests a strong affinity between the CoS_2_ adsorption sites and MB molecules, promoting their effective retention on the material’s surface.

Furthermore, the value of 1/falling between 0 and 1 indicates a heterogeneous surface, where MB molecules can interact with adsorption sites of varying energy levels, thereby facilitating multilayer adsorption.^52^ This behavior is likely due to the physicochemical characteristics of CoS_2_, such as its irregular porous morphology, nonuniform pore size distribution, and the presence of surface functional groups, all of which may contribute to the effective immobilization of adsorbed molecules.

Moreover, the increasing values of *K*_F_ and *n* with rising temperature indicate that the adsorption process becomes advantageous at higher temperatures.^53^ Additionally, the experimental data showed excellent consistency with the Freundlich model, as evidenced by a high correlation coefficient (*R* ^2^ = 0.99), confirming that this model accurately describes the adsorption behavior of MB on CoS_2_ nanoparticles (Figure 10). Analysis of the Langmuir model revealed a theoretical maximum adsorption capacity (*q_m_*) of 128.20 mg/g at 298 K and 222.22 mg/g at 318 K, indicating that the adsorption of MB on CoS_2_ nanoparticles is an endothermic process favored by elevated temperatures. However, the calculated correlation coefficients from the Langmuir model were *R* ^2^ = 0.97 at 298 K and *R* ^2^ = 0.96 at 318 K, which are slightly lower than those obtained for the Freundlich model (Figure 10). This indicates that the adsorption process does not strictly conform to the Langmuir assumption of monolayer adsorption on a homogeneous surface.

**Figure 10:**
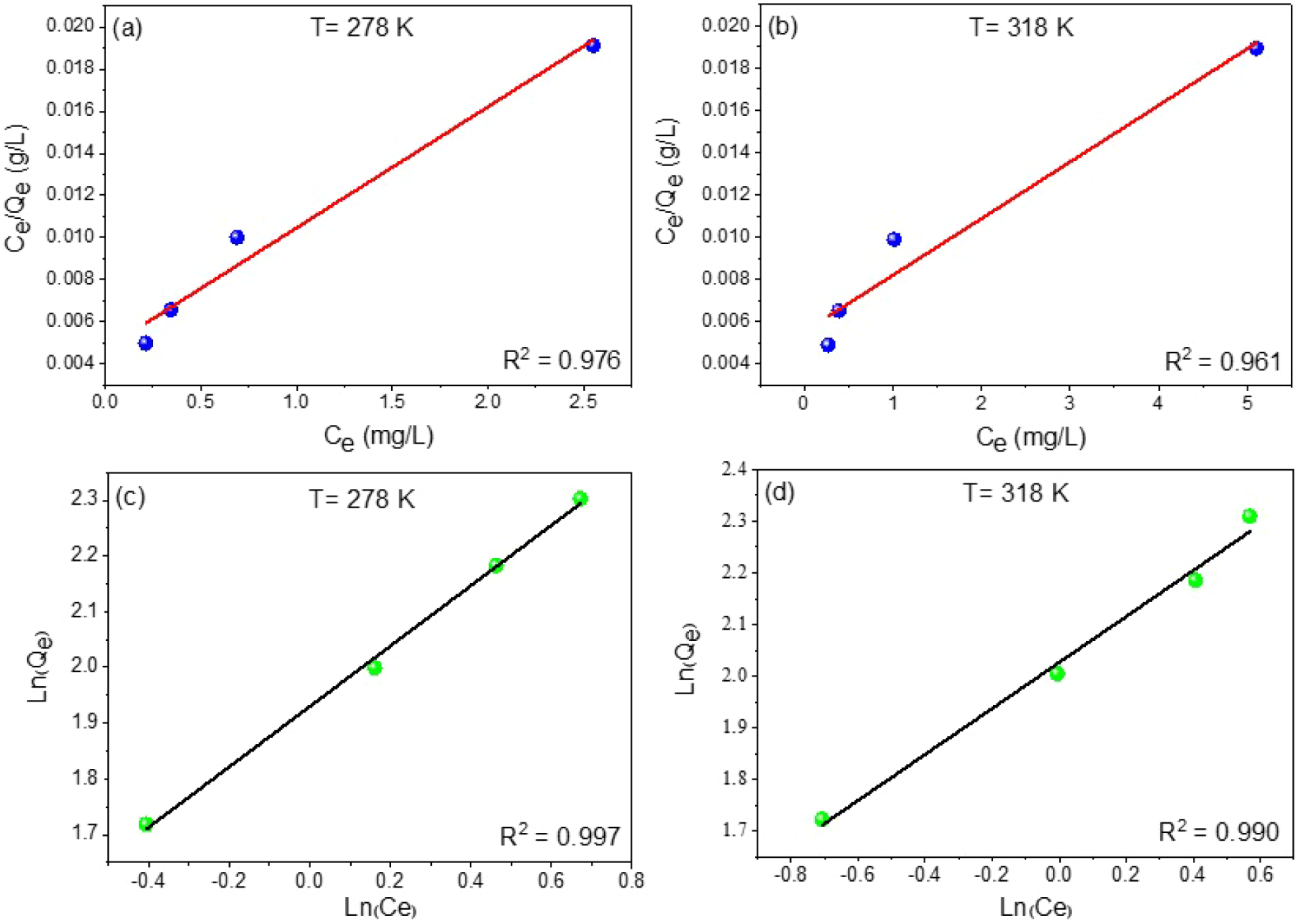
Langmuir (a and b) and Freundlich (c and d) isotherm plots for methylene blue (MB) adsorption onto CoS_2_ nanoparticles at 298 and 318 K.

Therefore, these findings indicate that the adsorption of MB onto CoS_2_ nanoparticles is better described by the Freundlich model, suggesting a mechanism dominated by heterogeneous surface interactions and multilayer adsorption, rather than monomolecular adsorption on uniform sites.^54,55^ Table 3 summarizes the parameters of the Langmuir and Freundlich isotherms for MB adsorption on CoS_2_ NPs at 298 and 318 K.

**Table 3:** Langmuir and Freundlich Isotherm Parameters for MB Adsorption onto CoS_2_ Nanoparticles at 298 and 318 K

	Isotherm constants					
Model	*T* = 298 K			*T* = 318 K		
Langmuir	*Q* (mg/g)	*K*_L_ (L/mg)	*R* ^2^	*Q* (mg/g)	*K*_L_ (L/mg)	*R* ^2^
	128.2	2.228	0.971	222.22	0.109	0.961
Freundlich	1/*n*	*K*_F_ (mg/g)	*R* ^2^	1/*n*	*K*_F_ (mg/g)	*R* ^2^
	0.541	6.891	0.997	0.445	7.588	0.99

The stability and reusability of the CoS_2_ adsorbent are critical parameters for evaluating its effectiveness in practical wastewater treatment applications. In this study, the CoS_2_ adsorbent synthesized at 150 °C was reused for six consecutive cycles under the optimal conditions for MB removal. Prior to each reuse, the material was thoroughly rinsed with distilled water and ethanol solvents, and lastly dried under the previously described conditions.

Figure 11 illustrates the evolution of the degradation rate (R%) of MB with the number of adsorption cycles. As shown, the removal efficiency slightly decreases with each cycle. Specifically, from the first to the second cycle, the degradation rate decreased marginally by only 0.02%. Between the second and sixth cycles, a cumulative decrease of approximately 4.22% was observed.

**Figure 11:**
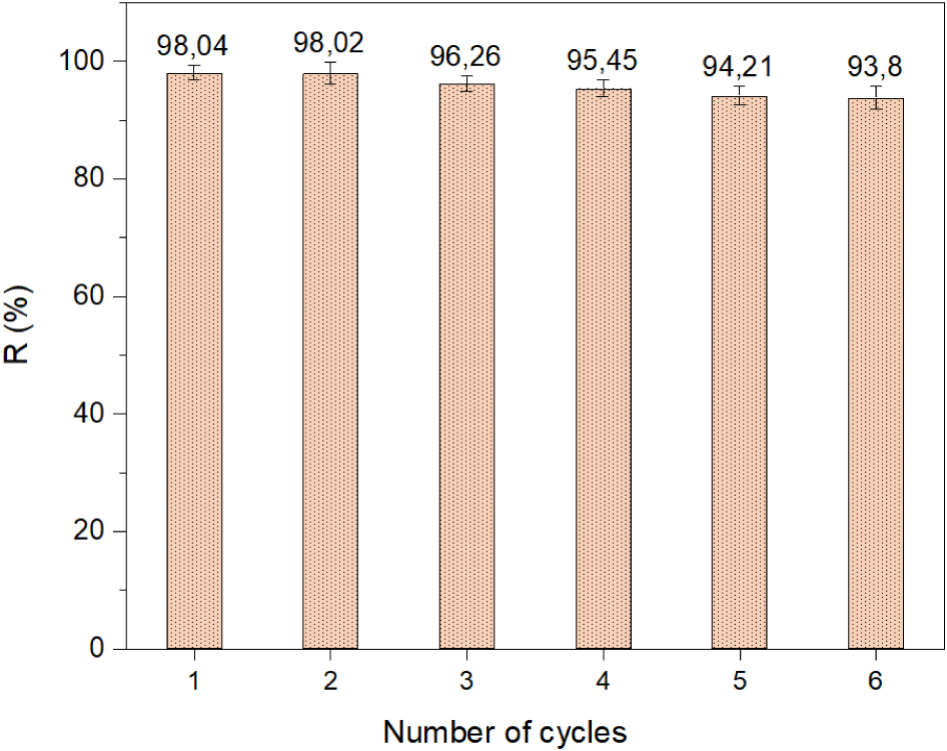
Evolution of the MB removal rate (%) over multiple cycles during the recyclability test of the CoS_2_ adsorbent.

These results demonstrate the excellent stability and reusability of the CoS_2_ adsorbent, supporting its potential for repeated use in MB removal via adsorption with only a minimal decline in performance over multiple cycles. To further investigate the morphological stability of the adsorbent after multiple reuses, SEM analysis was performed on the CoS_2_ sample recovered after six adsorption–desorption cycles. As shown in Figure 12, the grains of CoS_2_ nanoparticles exhibit clear agglomeration, resulting in a morphology with reduced porosity and a decline in the effective surface area. This observation is consistent with the slight decrease (∼4%) in MB removal efficiency, confirming that limited aggregation occurs during the recycling process.

**Figure 12:**
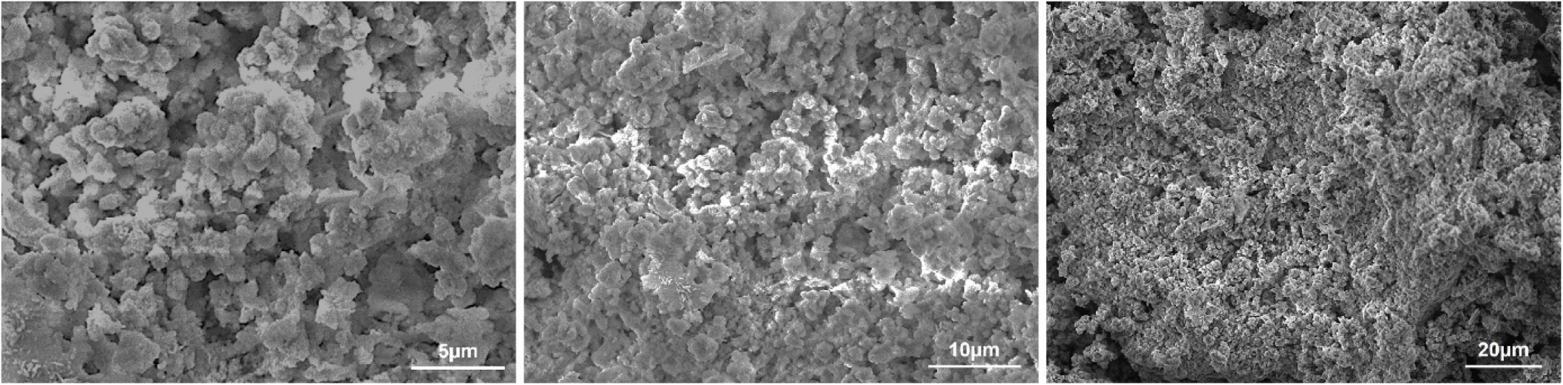
SEM micrographs of CoS_2_ nanoparticles after six adsorption–desorption cycles at different magnifications.

## 4 Conclusion

In summary, the characterization techniques, including SEM, EDX, XRD, and FTIR, confirmed the successful hydrothermal synthesis of pure cobalt disulfide (CoS_2_) nanoparticles with 3D nanostructures at 150 °C, 180 °C, and 210 °C. Among these, the sample synthesized at 150 °C exhibited the best adsorption performance owing to its smaller grain size and higher surface area. The adsorptive performance of CoS_2_ nanoparticles was investigated by using methylene blue (MB) as a model organic dye. The optimized CoS_2_ adsorbent achieved a maximum MB removal efficiency of approximately 98% within 2 h under alkaline conditions (pH 12). Kinetic studies demonstrated that the adsorption process follows the pseudo-second-order model, revealing a chemical sorption mechanism. Isotherm studies confirmed that the adsorption behavior is effectively represented by the Freundlich model, indicating multilayer adsorption on a heterogeneous surface. The process was endothermic, and the adsorption capacity increased with temperature. The stability and reusability of the CoS_2_ adsorbent were confirmed by recyclability tests, which showed only a minor decrease (∼4%) in the removal efficiency over six cycles. These results highlight the potential of hydrothermally synthesized CoS_2_ nanoparticles as cost-effective, efficient, and reusable adsorbents for wastewater treatment applications.

## Data Availability

The raw data supporting the findings of this study are not publicly available, as they are part of an ongoing study and will be used in future publications. All relevant processed data required to support the conclusions of this work are included within the article. The raw data are available from the corresponding author upon reasonable request.
